# Nerve growth factor promotes the proliferation of Müller cells co-cultured with internal limiting membrane by regulating cell cycle via Trk-A/PI3K/Akt pathway

**DOI:** 10.1186/s12886-019-1142-x

**Published:** 2019-06-17

**Authors:** Luyi Zhang, Xiaoxia Li, Xiaoqin Lin, Miaoqin Wu

**Affiliations:** Department of Ophthalmology, Zhejiang Provincial People’s Hospital, People’s Hospital of Hangzhou Medical College, 158 Shangtang Road Hangzhou, Zhejiang, 310014 China

**Keywords:** Nerve growth factor, Internal limiting membrane, Müller cells, Cell proliferation, Proliferation-related pathway, Cell cycle

## Abstract

**Background:**

Nerve growth factor (NGF), produced by Müller cells, and internal limiting membrane (ILM) have fundamental roles in the development of full-thickness macular hole (FTMH). However, the potential crosstalk between NGF and ILM in FTMH is unclear. This study aimed to explore the mechanism and effects of NGF on the proliferation of Müller cells co-cultured with ILM.

**Methods:**

Primary Müller cells and ILM from New Zealand rabbits were extracted and authenticated with specific staining. Müller cells co-cultured with or without ILM were exposed to NGF and then analysed. Müller cell viability was estimated using cell counting kit-8. Cell cycle analysis was performed by flow cytometry. The levels of cell cycle-related gene were detected using qRT-PCR. The TrK-A/Akt signal axis and downstream signaling cascades such as p21, CyclinE, CDK2, CyclinD1, and CDK4 were investigated by western blotting.

**Results:**

ILM treatment alone induced the proliferation of Müller cells following the promotion of phosphorylated Akt, while growth of Müller cells was enhanced by activation of the Trk-A/Akt pathway under the stimulation of NGF or NGF + ILM. Additionally, the ratio of S-phase cells was increased, while G2-phase cells decreased upon the treatment with either ILM or NGF alone, or with NGF + ILM co-treatment. Cell cycle-related genes such as CyclinD1, CyclinE, CDK2, and CDK4 were all upregulated, but p21 expression was downregulated in the presence of NGF, ILM, or NGF + ILM. There was an additive effect on cell proliferation and cell cycle in the group of Müller cells exposed to NGF co-cultured with ILM compared with either NGF or ILM treatment alone. However, both K252ɑ (inhibitors of Trk-A) and LY294002 (inhibitor for Akt) counteracted the effect of NGF or NGF + ILM on the protein levels of Trk-A, Akt, CyclinD1, CyclinE, CDK2, and p21.

**Conclusions:**

Müller cells co-cultured with ILM or NGF promoted cell proliferation by regulating cell cycle-correlated proteins via the PI3K/Akt pathway. ILM + NGF further amplified the PI3K/Akt signaling pathway by binding to Trk-A, leading to more cell growth. This study provides new insight into the potential mechanism of NGF-mediated proliferation of Müller cells co-cultured with or without ILM, which may have considerable impact on therapies for FTMH.

**Electronic supplementary material:**

The online version of this article (10.1186/s12886-019-1142-x) contains supplementary material, which is available to authorized users.

## Background

Full-thickness macular hole (FTMH), a defect in the fovea of the retina, is a major cause of central vision deterioration [1, 2]. Incidence of macular hole (MH) is 0.1 to 0.3%, of which 11.7% are bilateral [3–5]. Surgical intervention in the form of vitrectomy has become the main mode of treatment for FTMH^6^. The surgery has benefited a majority of eyes with FTMH by promoting anatomical closure. The rate of anatomic success has been improved through the use of wound-healing adjuvants, such as transforming growth factor-beta 2, which promotes fibroblast proliferation and collagen synthesis [7]. Other studies reported 100%/88% anatomic success rates in 11 eyes using serum/autologous platelet concentrate as the adjuvants, respectively [8, 9]. However, macular hole closure-related factors and the extracellular signals that modulate and control these processes are not fully understood.

Recently, new evidence implies that active and dynamic Müller cells are involved in the pathogenesis of idiopathic FTMH and subsequent epiretinal membrane (ERM) formation [10]. Müller cells are a special type of neurogliocytes that span the entire depth of the neural retina [11]. They are anatomically and functionally essential for retinal development and homeostasis, serving as supporting cells for the neurons of the retina [12]. Research has shown that the structure and function of the end feet of Müller cells are closely related to retinal ganglion cells [13]. Therefore, active regulation of Müller cells may facilitate development of neural axons in the retina and subsequently provide protection from FTMH disease.

Internal limiting membrane (ILM), the basement membrane of Müller cells, is produced by Müller cells during the development of the retina under normal conditions [9]. However, in patients with macular hole (MH), the ILM provides extracellular matrix for cellular migration and contraction, causing the enlargement of MH accompanied by necrosis of Müller cells [14]. It has been confirmed that peeling of ILM has a therapeutic effect for traumatic MH [15, 16]. ILM also has a fundamental role in the development and angiogenesis of the retina [17]. Michalewska and associates have used the inverted ILM flap technique to improve the closure rate of large macular holes [18]. Recently, Novelli et al.[18] transplanted autologous ILM into the MH so that it successfully stimulated Müller cell proliferation and promoted the progression of MH closure, suggesting that autologous transplant of ILM might improve the symptoms of MH by activating Müller cell growth.

Nerve growth factor (NGF), a classic neuroprotective factor, increases vascular endothelial growth factor (VEGF) expression and promotes cell proliferation via activating ERK1/2 and AKT signaling through binding to its receptor Trk-A [14]. NGF, produced by Müller cells, also plays a critical role in retinal neovascularization [20], indicating that ILM and NGF may cross-react during retinal development. However, whether there is correlation between NGF and ILM in promoting proliferation with Müller cells remains unknown.

The effects and the mechanism of NGF on the proliferation of Müller cells co-cultured with or without ILM are illustrated in the present study. Overall, the study provided new insight for the potential mechanism of the NGF-mediated proliferation of Müller cells co-incubated with ILM, which might be developed as a neuroprotective agent for the treatment of FTMH.

## Methods

### Materials

Primary antibodies against CyclinD1, CyclinE, CDK2 and CDK4 were obtained from Lifespan International Inc. (LS-C425816, US). Primary antibodies against Trk-A, p-Trk-A, Akt, p-Akt and p21 were purchased from Abcam (UK). NGF was from PEPROTECH (US). The inhibitor for Trk-A, K252ɑ, and the inhibitor for Akt, LY294002, were purchased from MCE (US).

### Isolation of primary Müller cells and ILM

New Zealand rabbits were treated in a humane fashion consistent with ARVO guidelines [21]. The animal care procedures were reviewed and approved by the Laboratory Animal department of Hangzhou Medical College in this study. For Müller cell isolation, New Zealand rabbits were sacrificed with an overdose injection of sodium pentobarbital(Merck, GER) and retinas were isolated after removing the anterior segment and vitreous body. Then they were cut into approximately 1 mm^3^ pieces and digested with trypsin followed by filtration. After transient centrifugation, cells were cultured in complete 1640 medium (Hyclone, US) with 20% fetal bovine serum (FBS, Gibco, US) in a humidified environment of 5% (vol./vol.) CO_2_ at 37 °C for 12 h. Cells were washed twice with D-Hank’s buffer to remove the floating retinal neurons and vascular endothelial cells, and then replenished with complete medium. After screening for 3–4 generations, Müller cells were verified with vimentin and glutamine synthetase (GS) immunofluorescent staining (Abcam, UK). Each experiment used the same cells at a different passage (Additional file 1: Figure S1). Next, internal limiting membrane was peeled from New Zealand rabbits using disposable syringe needles. First, a crochet hook made from syringe needles was used to provoke the edge of ILM. Then, the boundaries of ILM peeling were determined by observing reflection changes of the retinal surface in the front and back of ILM, and ILM were grasped and peeled using forceps. After peeling, ILM was placed in 1640 medium and co-cultured with Müller cells.

### Cell treatment

Primary Müller cells were cultured to the third generation in complete medium (1640 with 20% FBS). Then, cells were used to perform experiments at passage 4, with passages between the first and last experiments not exceeding five generations. For construction of the co-culture system, internal limiting membrane was placed in 1640 medium for 24 h followed by incubation in 12-well plates, and then Müller cells (0.5 × 10^5^ cells/well) were seeded in the plate with 20% FBS. According to previous reports and our preliminary experiments (Additional file 2: Figure S2), 1 ng/ml NGF was used for the cell treatment [22]. For all groups of cells, the media were replaced with fresh media containing 1 ng/ml NGF to maintain the stated concentration of growth factor per day. For the inhibitor experiments, Müller cells+NGF, Müller cells+ILM, and Müller cells co-cultured with ILM exposed to NGF were pretreated with K252α (400 nM) and LY294002 (10 μM) for 48 h. Then, all groups were analysed by Western blot to evaluate the role of K252α and LY294002 in cell proliferation-related signaling transduction.

### CCK8 assay

For the CCK8 assay, Cell Counting Kit 8 (CCK8, Dojindo, Japan) was used according to the manufacturer’s instructions. ILM was placed in 1640 medium for 24 h followed by incubation in 96-well plates, then Müller cells were added to the plates at a density of 5 × 10^3^ cells/well and co-cultured for 24 h, 48 h, 72 h, and 96 h. At the appropriate time points (24 h, 48 h, 72 h, and 96 h), 10 μl of CCK8 reagent was added to each well and mixed uniformly. Then all cells were incubated for another three hours, and the optical absorbance at 450 nm was measured.

### Quantitative RT-PCR

Total RNA was isolated from all cells using TRIzol reagent (Roche, USA) according to the manufacturer’s protocol. 1 μg total RNA was reverse transcribed into cDNA with an All-in-One First-Strand cDNA Synthesis Kit (Transgene, China). All gene primer sequences including CyclinD1, CyclinE, CDK2, CDK4, and p21 are shown in Additional file 3: Table S1. In brief, the expression levels of the mRNAs were evaluated by real-time qPCR using SYBR Green reagent (Roche, Indianapolis, IN, USA). All samples were set up in five technical replicates, and each experiment was done at least three times. β-Actin served as the internal control. Calculation for gene relative expression was according to the 2^-∆∆Ct^ method.

### Western blot

Total cellular protein was extracted from different groups, and Western blot analysis was performed as previously described [23]. 30 μg of total protein lysates were separated by SDS-PAGE and the expression levels of different proteins were detected with specific primary antibodies. After incubation with peroxidase-conjugated secondary antibodies, ECL images were captured using x-ray film. The blot greyscale was quantified by using Quantity One analysis software (Bio-Rad). First, the optimized images were chosen to create lanes and analyse bands according to the software manual. The greyscale of targeted bands was normalized to the greyscale of β-actin, and relative greyscale was analyzed using SPSS software.

### Flow cytometry

ILM was placed in 1640 medium for 24 h followed by incubation in 6-well plates, then Müller cells were added into the plates at a density of 5 × 10^5^ cells/well and co-cultured, and cells were treated with 1 ng/mL NGF for 48 h. Then, cells were washed twice with ice-cold PBS buffer before digestion using trypsin without EDTA. Next, the cells were fixed with 1 mL 70% ethanol overnight. After transient centrifugation, cells were washed twice with ice-cold PBS and stained with 2 μL 50 μg/mL PI and 1 μL RNase A (100 μg/mL) dissolved in binding buffer for 30 min at 4°Cin darkness. Cell cycle was measured by flow cytometry (BD Biosciences), and the results were analyzed with Tree Star FlowJo software.

### Statistical analysis

All experiments were repeated at least three times. The data are reported as the mean ± SD. Statistical analysis on proliferation rate and cell cycle-related gene expression were performed using GraphPad Prism 5.0 software followed by one-way ANOVA and multiple comparisons tests. *P* < 0.05 was defined as statistically significant.

## Results

### NGF and NGF + ILM promoted cell proliferation of Müller cells by activating the Trk-a/PI3K/Akt pathway

**Fig. 1:**
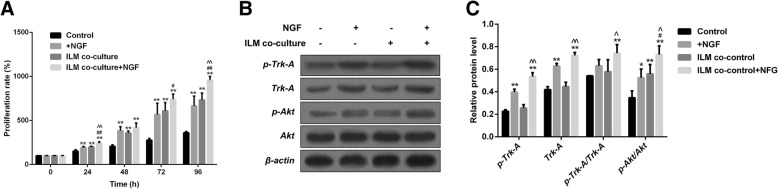
Effects of NGF, ILM and NGF + ILM on the proliferation of Müller cells. **a** CCK8 assay results of cell growth in different groups (Control, NGF, ILM, NGF + ILM). **P < 0.01, NGF/ILM/NGF + ILM vs Control at each time point; ^#^*P* < 0.05, ^##^*P* < 0.01, ILM + NGF vs NGF at each time point; ^^*P* < 0.01 ILM + NGF vs ILM at each time point. **b** The expression of p-Trk-A, Trk-A, p-Akt and Akt were evaluated by Western blot using specific primary antibodies in four different groups of Müller cells. **c** Quantitative analysis of protein levels of p-Trk-A, Trk-A p-Trk-A/rk-A and p-Akt/Akt in panel **b**. * indicates NGF/ILM/NGF+ILM vs Control; # indicates ILM+NGF vs NGF; ^^ indicates ILM+NGF vs ILM at each time point. *, ^, #, *P* < 0.05; **, ^^, ##, *P* < 0.01

Neurotrophic factors (NTFs) can promote the initial growth and development of neural retinal cells [24]. NGF and ILM play a crucial role in the development of neural retinal cells [25, 26]. Therefore, we surmised that NGF and ILM might participate in the process of Müller cell growth. To verify our hypothesis, Müller cells were exposed to NGF, ILM, and NGF + ILM. As shown by the CCK8 assay (Fig. 1a), in comparison to the control group, NGF, ILM, or NGF + ILM treatment dramatically enhanced the growth rate of Müller cells starting from 24 h, with the greatest effect seen in the NGF + ILM group (NGF vs control at 24, 48, 72, 96 h, *p* = 0.0061, 0.0032, 0.0042, and 0.0021; ILM vs control, *p* = 0.0023, 0.0078, 0.0097, 0.0086; NGF + ILM vs control, *p* = 0.0003, 0.0008, 0.0002, 0.0004). A higher proliferation rate was observed in the NGF + ILM group compared to the NGF-only group at 24 h (NGF + ILM vs NGF, *p* = 0.0051), 72 h (NGF + ILM vs NGF, *p* = 0.041) and 96 h (NGF + ILM vs NGF, *p* = 0.033). In comparison to the ILM-only group, the viability of Müller cells increased more obviously in the NGF + ILM group at 24 h (NGF + ILM vs ILM, *p* = 0.0041) and 96 h (NGF + ILM vs ILM, *p* = 0.0018). Collectively, these data suggest that the combination of NGF and ILM has a synergistic effect on Müller cell proliferation.

Previous work has verified that NGF promotes cell viability via PI3K/Akt through binding to Trk-A [14]. Thus, we thought that PI3K/Akt signaling transduction might be involved in NGF and NGF + ILM-mediated Müller cell proliferation. As indicated in Fig. 1b, once exposed to NGF, Trk-A and phosphorylated Trk-A were significantly upregulated in Müller cells, indicating that 1 ng/ml NGF was a suitable concentration to induce the activation of its receptor, Trk-A (Fig. 1b, second row). When co-cultured with ILM, upregulation of Akt phosphorylation, but not Trk-A activation, was observed in Müller cells, with an additive effect in the presence of NGF (Fig. 1b, last two rows). Accompanied by activation of Akt, Trk-A phosphorylation was enhanced with NGF treatment and showed more activation in co-treatment with NGF and ILM (Fig. 1b). Quantitative analysis also confirmed the variation tendency of Trk-A, p-Trk-A and p-Akt in panel B (Fig. 1c). Thus, we believe that NGF enhanced Müller cell proliferation, possibly via Trk-A/PI3K/Akt-mediated cell cycle acceleration, while ILM co-culture further amplified this effect through activating PI3K/Akt signaling independent of Trk-A. Our present findings clearly show that NGF and co-culture with ILM facilitate the proliferation of Müller cells, potentially involving Trk-A and PI3K/Akt pathways.

### NGF, ILM and NGF + ILM accelerated cell cycle progression of Müller cells

**Fig. 2:**
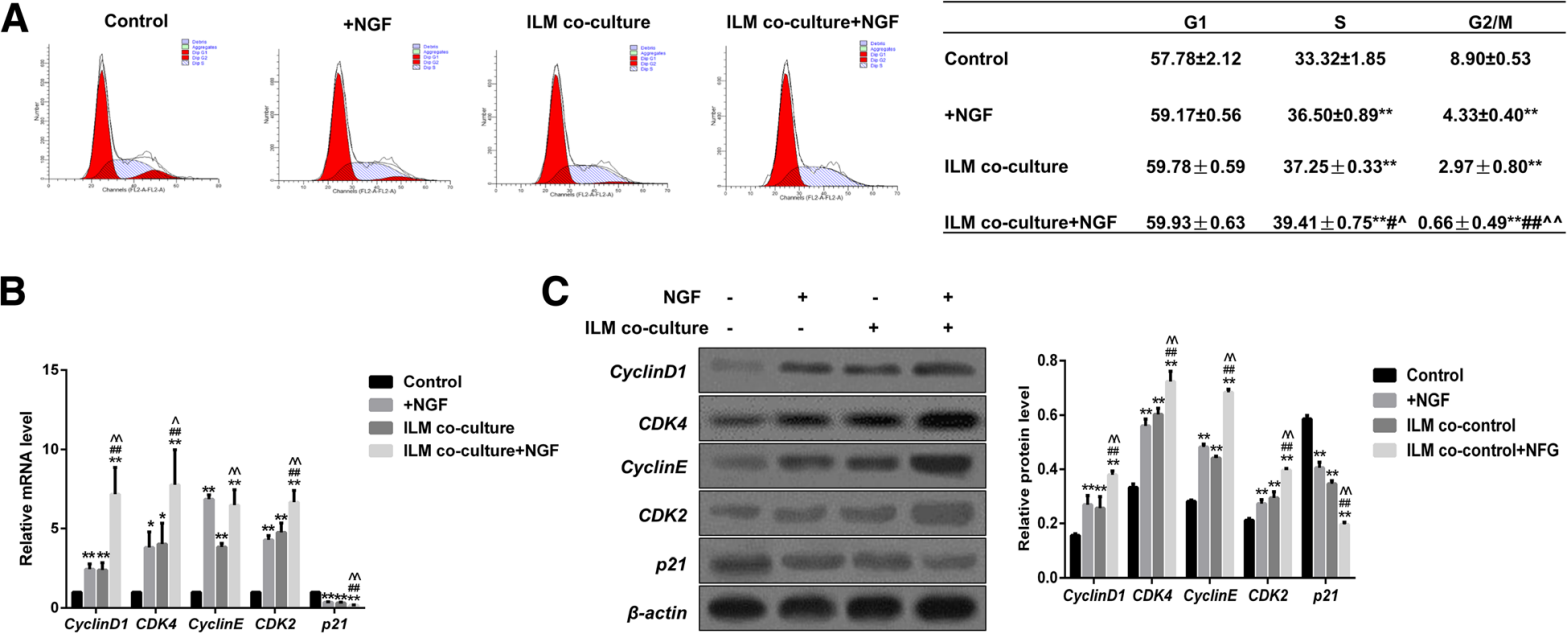
Effects of NGF, ILM and NGF + ILM on cell cycle progression of Müller cells. **a** The images of cell cycle analyses result in four different groups of Müller cells (upper panel), and the percentage of each phase (G1-M) is indicated (lower panel). Light blue indicates cell debris; light green indicates cell aggregates; red indicates G1-phase (left) and G2-phase (right) cells; and the oblique line indicates S-phase cells. Relative mRNA levels (**b**) and protein levels (**c**) of CyclinD1, CyclinE, CDK2, CDK4, and p21 in four different groups of Müller cells. *P < 0.05, **P < 0.01 NGF/ILM/NGF + ILM vs Control; ^#^*P* < 0.05, ^##^ *P* < 0.01, ILM + NGF vs NGF; ^^^*P* < 0.05, ^^*P* < 0.01 ILM + NGF vs ILM

Since NGF and ILM had a strong proliferation-promotion effect on Müller cells, we next explored whether NGF and ILM-mediated proliferation enhancement was involved in the alteration of cell cycle progression. The result showed that both NGF and co-culture with ILM treatment could prevent S-phase cells from entering G2/M in Müller cells. Moreover, when Müller cells were co-cultured with ILM + NGF, more cells were in S-phase and fewer cells were in G2/M-phase than in cultures treated with NGF or ILM only (Fig. 2a). It has been confirmed that CyclinD1-CDK4 and CyclinE-CDK2 are the key kinase complexes in the progression of cell cycle from G1 to S phase [27]. Therefore, we evaluated these kinase activities in our model. As observed in Fig. 2b and c, transcriptional and protein levels of key cell cycle-related genes, including CyclinD1, CyclinE, CDK2 and CDK4, all increased in the presence of NGF and ILM. Co-treatment with NGF and ILM increased this effect, while *p21* (cyclin-dependent kinase inhibitor 1) decreased by comparison (Fig. 2b-c). In brief, these data show that NGF and ILM can affect the cell cycle of Müller cells via increasing the S-phase cell population.

### Trk-a/PI3K/Akt signaling pathway was required in the process of NGF- and ILM-induced cell cycle and proliferation promotion

**Fig. 3:**
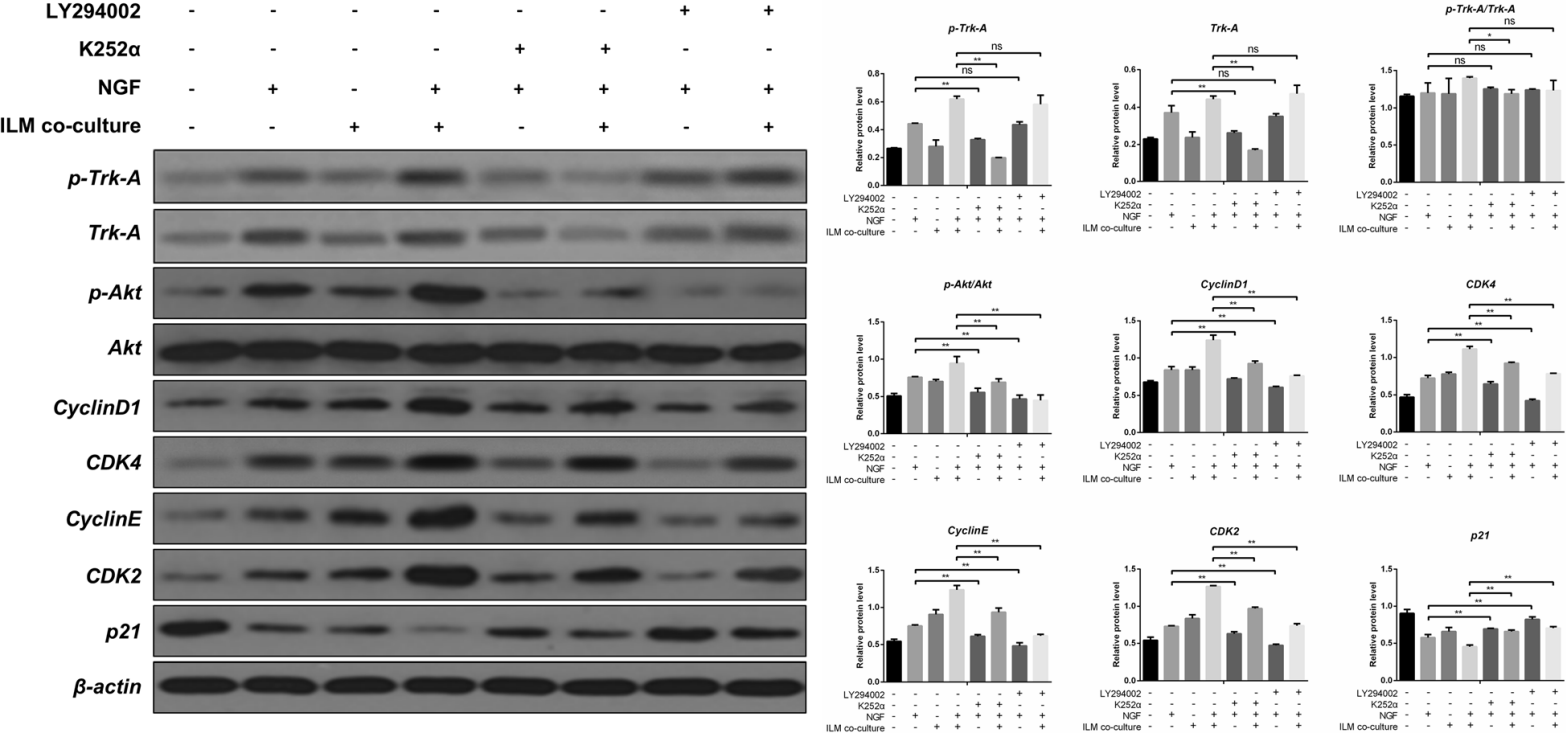
The impacts of K252α and LY294002 on the levels of several key cell cycle-related proteins. Protein levels of Trk-A, phosphorylated Trk-A, Akt, phosphorylated Akt, CyclinD1, CyclinE, CDK2, CDK4, and p21 in eight different groups of Müller cells were measured by western blotting assay. K252α indicates the inhibitor of Trk-A and LY294002 indicates the inhibitor of Akt

To determine whether Trk-A and PI3K/Akt activation induced the cell cycle under NGF or ILM treatment alone or with NGF + ILM co-treatment, the western blot assay was performed on cell cycle-related proteins in the presence of Trk-A and Akt inhibitors. Similar to the above data, activation of Trk-A and Akt, as well as expression of CyclinD1, CyclinE, CDK2, and CDK4 were promoted, whereas p21 level decreased in treatment with NGF or ILM alone (Fig. 3a, second and third row). Once Müller cells were co-cultured with ILM, the role of NGF on proliferation- and cell cycle-related signaling molecules increased (Fig. 3a, fourth row). However, in the presence of inhibitors of Trk-A (K252ɑ) and Akt (LY294002), NGF induced the increase of Trk-A, Akt, CyclinD1, CyclinE, CDK2, and CDK4, and the decrease of p21 was markedly neutralized (Fig. 3a, fifth and seventh row). Similarly, Trk-A and Akt inhibition using K252ɑ and LY294002 significantly counteracted the regulatory role of NGF on cell proliferation and cell cycle-related signaling molecules in Müller cells co-cultured with ILM (Fig. 3a, sixth and eighth row).

## Discussion

FTMH is a vital cause of central visual loss worldwide, with an estimated incidence of 7.8 persons/100,000 population per year [5]. Treatment strategies for FTMH previously consisting of relieved tractions by the thorough removal of the vitreous humor with gas tamponade (first described by Kelly and Wendel), now consist of ILM peeling and removal of proliferations at the retinal surface combined with indocyanine green injection [6, 28].

ILM peeling performs a beneficial function in speeding the rate of anatomical closure of MH [29, 30].Among large MHs with diameters greater than 500 μm, fewer than 60% of them were closed after the first surgery, and 19 to 39% were flat-open [18]. Several modifications have been investigated to solve problems in the surgical treatment, including the use of an inverted ILM flap technique [18] and autologous ILM transplantation [19]. Peeled ILM brings adhered cellular remnants of Müller cells into the bare area of the MH to serve as a scaffold for glial cell proliferation. Active glial cell proliferation often results in scar formation, which is not good for visual acuity recovery and metamorphosis remission; faster MH closure could reduce the problem [18, 31]. In fact, though a large percentage of patients’ macular hole has fully healed after surgery, the best corrected visual acuity remains unsatisfactory.

Müller cells, the major glia cell of the retina, have been regarded as an important source of various cytokines, such as NGF and VEGF, which have been confirmed to regulate the proliferation of Müller cells mainly through the Trk-A signaling pathway [20, 32, 33]. Though emerging research has indicated that NGF plays an essential role in the proliferation of Müller cells, the underlying mechanism is not clear. In this study, we demonstrated that NGF or ILM alone, or the two together, can enhance the proliferation ability of Müller cells. These results offered novel evidence that ILM co-cultured with Müller cells distinctly promotes cell proliferation, especially in the presence of NGF. Moreover, with flow cytometry and western blot analysis, we found that NGF and ILM had an additive effect on the cell cycle progression of Müller cells, implying that NGF and ILM had additive action on Müller cells proliferation through the cell cycle.

**Fig. 4:**
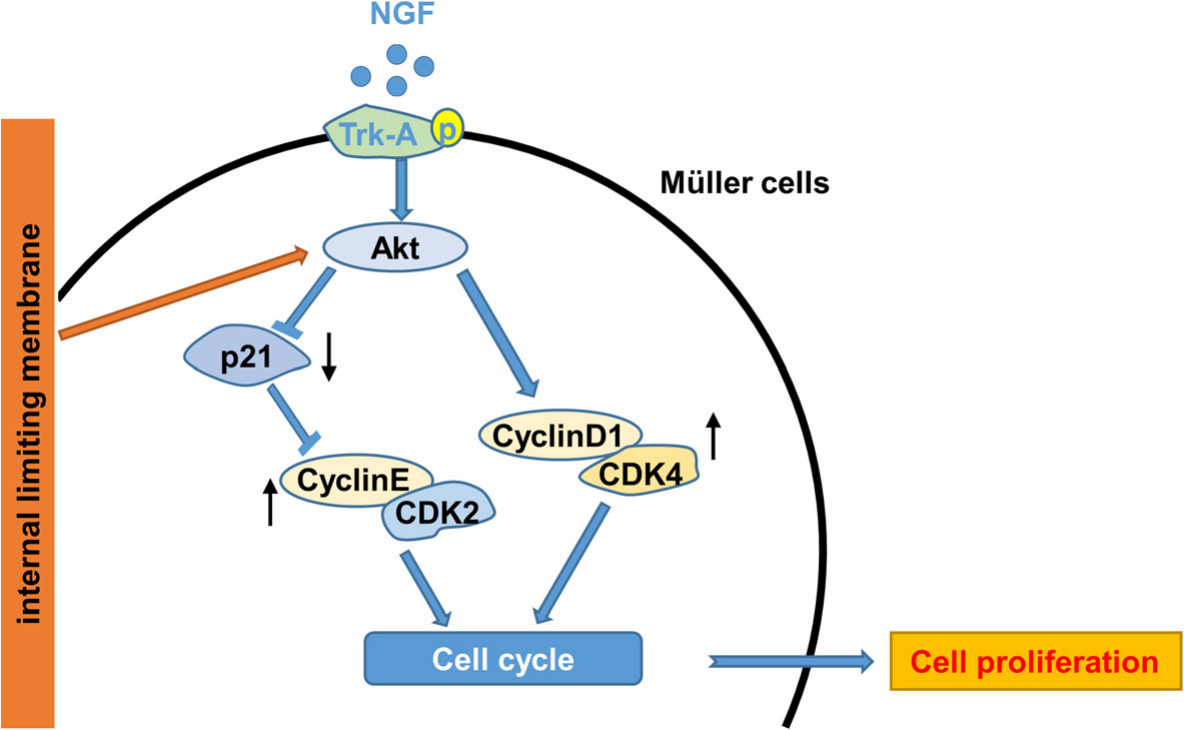
The mechanistic diagram of NGF and ILM-mediated proliferation promotion by regulating cell cycle of Müller cells. ILM co-culture enhanced the activity of Akt independent of Trk-A and the downstream p21-CyclinE-CDK2 and CyclinD1-CDK4 signaling pathways, leading to cell cycle and cell growth enhancement. NGF exposure mediated the activation of Trk-A and further promoted the upregulation of the PI3K/Akt pathway followed by the increase of CyclinD1-CDK4, and by the decrease of p21 and CyclinE-CDK2 kinase complex activities, which then induced cell cycle progression and cell growth promotion in Müller cells

It is known that NGF can reduce the apoptosis of photoreceptors from retinal detachment injury, and has a protective effect on Müller cells to relieve retinal gliosis in rats via the Trk-A signaling pathway [34]. Intravitreal injection of NGF has been used in the Royal College of Surgeons (RCS) rat model of retinitis pigmentosa, and the investigators believed the protective effect was associated with an increase in Trk-A and activated pTrk-A levels [35]. However, the mechanism by which NGF regulates the proliferation of Müller cells remains unknown. The binding of NGF to its receptor, Trk-A, could mediate the activation of the downstream PI3K/Akt signaling pathway. In our study, NGF exposure mediated the activation of Trk-A and further promoted the upregulation of PI3K/Akt signaling transduction followed by the increase of CyclinD1-CDK4. At the same time, p21 and CyclinE-CDK2 kinase complex activities decreased, which then induced cell cycle progression and cell growth promotion in Müller cells. ILM co-culture promoted the activity of Akt independent of Trk-A and the downstream p21-CyclinE-CDK2 and CyclinD1-CDK4 signaling pathway, leading to cell cycle and cell growth enhancement. Thus, based on our study, we believe that ILM could act as a supportive extracellular matrix, promoting the activation of intracellular PI3K/Akt signaling transduction. When Müller cells are co-incubated with ILM, NGF-mediated signal transduction and proliferation-promotion effects are robustly amplified (Fig. 4).

## Conclusions

In the present study, we found that either NGF alone or the combination of NGF and ILM could regulate cell cycle via Trk-A/PI3K/Akt pathways, followed by proliferation promotion of Müller cells. Based on our findings, it is possible to improve the outcome of FTMH surgeries through ILM transplantation combined with NGF treatment. Therefore, our investigations provide the underlying regulation mechanism of NGF and ILM on Müller cell proliferation, which may potentially provide new methods for the treatment of FTMH.

## Additional files

Additional file 1: **Figure S1.** Immunofluorescence identification of GS and vimentin in Müller cells (100×). (TIF 4354 kb)

Additional file 2: **Figure S2.** Effects of NGF on the concentration of L-Glutamine. (A) The contents of L-Glutamine at different points of time after treated with NGF(1ng/ml). (B) The contents of L-Glutamine at different concentration of NGF after treated for 48 h. *P<0.05, **P<0.01 vs. 0 group; (TIF 325 kb)

Additional file 3: **Table S1.** Primers sequences of cell cycle-related genes. (DOC 29 kb)

## Data Availability

All data generated and analysed during this study are included in this published article and its Additional files.

## Abbreviations

CCK8: Cell Counting Kit 8
CDK: Cyclin-dependent kinase
ERM: Epiretinal membrane
FBS: Fetal bovine serum
FTMH: Full-thickness macular hole
ILM: Internal limiting membrane
MH: Macular hole
NGF: Nerve growth factor
NTFs: Neurotrophic factors
RCS: Royal College of Surgeons
VEGF: Vascular endothelial growth factor
