# Supplementary figures and images for: Nerve growth factor promotes the proliferation of Müller cells co-cultured with internal limiting membrane by regulating cell cycle via Trk-A/PI3K/Akt pathway
Source: BMC Ophthalmol. 2019 Jun 17;19:130. doi: 10.1186/s12886-019-1142-x (PMC6580575; doi:10.1186/s12886-019-1142-x)

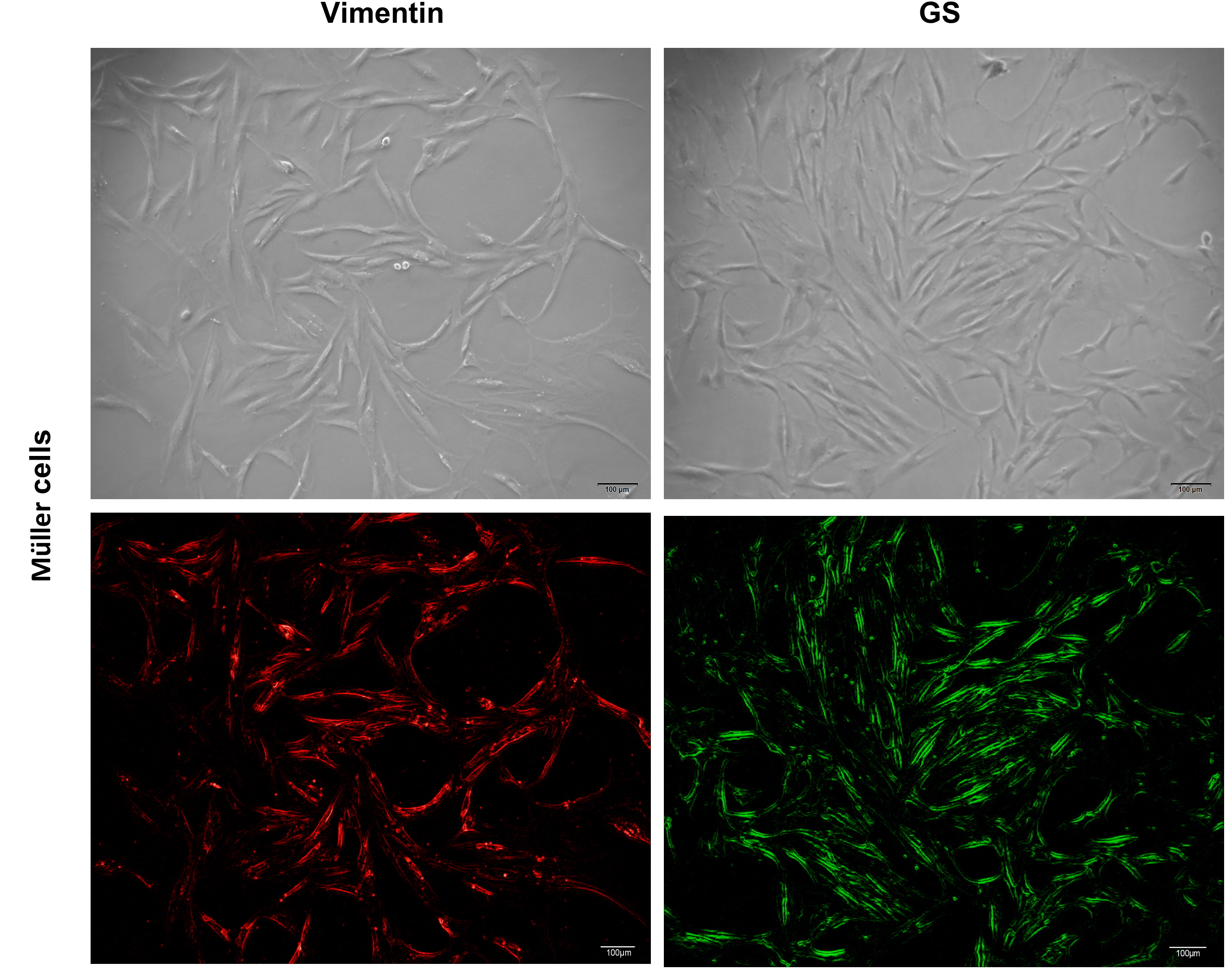

Supplement: Supplementary file 1 — Figure S1. Immunofluorescence identification of GS and vimentin in Müller cells (100×). (TIF 4354 kb) [file 12886_2019_1142_MOESM1_ESM.tif]

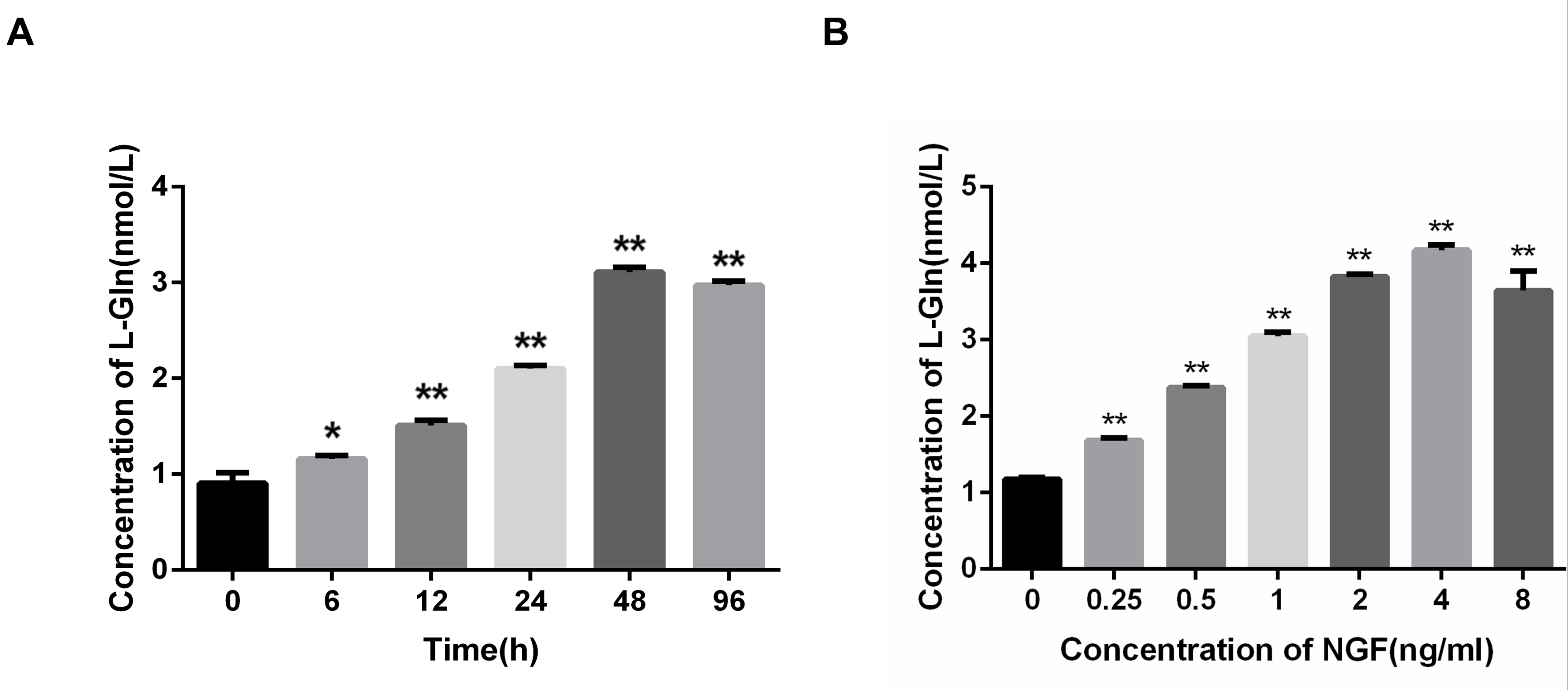

Supplement: Supplementary file 2 — Figure S2. Effects of NGF on the concentration of L-Glutamine. (A) The contents of L-Glutamine at different points of time after treated with NGF(1ng/ml). (B) The contents of L-Glutamine at different concentration of NGF after treated for 48 h. *P<0.05, **P<0.01 vs. 0 group; (TIF 325 kb) [file 12886_2019_1142_MOESM2_ESM.tif]
