# Supplementary material for: Nerve growth factor promotes the proliferation of Müller cells co-cultured with internal limiting membrane by regulating cell cycle via Trk-A/PI3K/Akt pathway
Source: BMC Ophthalmol. 2019 Jun 17;19:130. doi: 10.1186/s12886-019-1142-x (PMC6580575; doi:10.1186/s12886-019-1142-x)
Supplement: Supplementary file 3 — Table S1. Primers sequences of cell cycle-related genes. (DOC 29 kb) [file 12886_2019_1142_MOESM3_ESM.doc]

**Table S1. Primers sequences of cell cycle-related genes**

| **Genes** | **Sequences** |
| --- | --- |
| *CyclinD1* | Forward:5’-3’ TCAAGTGCGTGCAGAAGGAG  Reverse:5’-3’ GGAGTTGTCGGTGTAAATGC |
| *CyclinE* | Forward:5’-3’ ACACCCTCTTCTGCAGCCTA  Reverse:5’-3’ ATCTCGTCCCCTGAACAAGC |
| *CDK2* | Forward:5’-3’ CCACCCCAATATTGTCAAGC  Reverse:5’-3’ AGTCGGCTAACTTGATGGAG |
| *CDK4* | Forward:5’-3’ GGAAATGCTGACTTTCAGCC  Reverse:5’-3’ CTTCCTTGTGGAGGTAAGAG |
| *P21* | Forward:5’-3’ GCGCTGGAACTTCGACTTTG  Reverse:5’-3’ TTTCCGACCCTGAGAGGTA |
